# Motivating Quit Attempts Through Gamification: A Systematic Review of Digital Game-Based Interventions for Smoking Cessation

**DOI:** 10.7759/cureus.93399

**Published:** 2025-09-28

**Authors:** Spandita Das, Kunal Jha, Ipseeta Menon, Anukampa Senapati, Rohini Parui, Anwesha Mishra

**Affiliations:** 1 Public Health Dentistry, Kalinga Institute of Dental Sciences (KIDS) Kalinga Institute of Industrial Technology (KIIT) Deemed to be University, Bhubaneswar, IND; 2 Public Health, Maharaja Krishna Chandra Gajapati (MKCG) Medical College and Hospital, Berhampur, IND

**Keywords:** behavior therapy, gamification, motivational intervention, smoking, tobacco cessation

## Abstract

Tobacco use remains a leading cause of preventable disease and death worldwide. While quitting significantly reduces health risks, many individuals struggle to quit using traditional methods. With increasing access to mobile technology, gamified digital interventions have emerged as a novel approach to support smoking cessation. The aim of the review was to evaluate the effectiveness of gamification-based digital interventions in promoting smoking cessation, focusing on user engagement, abstinence rates, motivation, and implementation challenges. A systematic literature review was conducted using PubMed, Scopus, and Web of Science, guided by the population/patient/problem, intervention, comparison, and outcome (PICO) framework, to identify English-language articles published between January 2015 and July 2025 on gamified digital interventions for smoking cessation. A total of 136 articles were initially retrieved, out of which eight met all eligibility criteria and were included in the final review. Study selection followed the Preferred Reporting Items for Systematic Reviews and Meta-Analyses (PRISMA) guidelines, and data extraction was done independently by two reviewers. The quality of included studies was assessed using the National Institutes of Health (NIH) tool checklist for controlled intervention studies. Out of 136 articles, eight met the inclusion criteria and were reviewed in depth. The studies highlighted five key areas: user engagement, quit rates, motivation, user satisfaction, and implementation challenges. Gamified tools, like virtual pets, leaderboards, and challenges, boosted engagement and motivation but didn’t always lead to higher quit rates. Some apps, like SCAMPI and QuitIT, showed promising results, while others had mixed outcomes. Gamification can make quit-smoking apps more fun and motivating, helping people stay engaged and confident in their quit attempts. However, most studies showed only short-term benefits, and the evidence for long-term quitting is still weak.

## Introduction and background

The tobacco epidemic constitutes a significant public health threat globally, accounting for over seven million deaths each year, in addition to disability and prolonged suffering from ailments associated with tobacco use. Tobacco usage in any form is detrimental, and there is no amount of tobacco exposure that is considered safe [1]. Tobacco use is one of the primary causes of oral cancer, a severe and expanding global issue, and smoking or chewing tobacco can have a major negative impact on oral health as well as overall health [2]. In 2005, the World Health Organization Framework Convention on Tobacco Control (WHO FCTC) came into force, using the international law to reduce tobacco use. It attempts to address a few of the main contributors of that epidemic, including intricate elements that have an impact across borders, like liberalization of trade and direct foreign investment, tobacco sponsorship, promotion, and marketing abroad, and the illegal trade in tobacco goods [3].

Cessation of tobacco use reduces the risk of cardiovascular and pulmonary illnesses, numerous cancers, and early mortality [4]. The Centers for Disease Control and Prevention (CDC) believe that smoking cessation programs reduce premature death rates while also improving quality of life. People who have smoked in the past might expect to live an additional ten years after quitting [5].

Considering the potential reach and ease, smartphone-delivered tobacco cessation support presents a strong substitute for conventional professional therapies. Apps for quitting tobacco consumption in particular have become more well-liked because of their cost-effectiveness and immediate help [6]. As a supplement to conventional cessation services, mobile health (mHealth) apps offer a new technology with enormous potential for behavior modification [7]. Smokers’ motivation and willingness to cessation will be strengthened if smoking therapies are presented as quick, game-based activities. Game mechanics, which combine entertaining and incentive techniques, provide instant rewards and are based on findings from behavioral psychology. Effective reinforcement of desired behaviors can be achieved by well-designed games that offer positive reinforcement and criticism in addition to rewarding accomplishments [8].

The issues of inadequate knowledge and utilization of smoking cessation programs may be resolved by smartphone-based smoking cessation interventions [9]. Game-based interventions may also help alleviate cravings through additional mechanisms, such as redirecting attention or serving as an effective coping strategy [10]. Such interventions can be tailored to reflect youth culture, leverage peer dynamics, and accommodate diverse motivations and smoking patterns, thereby increasing engagement and retention among young smokers [11]. One of the core objectives of many well-known behavior modification strategies is motivation. Gamification, which tracks individual actions and engages users in goal-chasing activities while showing progress and feedback through individualized information apps, is one potential solution to the communication and motivational problems in the healthcare industry [12].

Gamification which is the integration of game design elements into non-game settings has shown potential in boosting user engagement within mHealth platforms and encouraging positive behavior change. Specifically, in smoking cessation efforts, mobile apps that use gamified features like education and progress monitoring have demonstrated increased user interaction and involvement [13]. These approaches have the potential to significantly increase smoking cessation rates [14].

Because of the superior accessibility, worldwide reach, and sophisticated processing power, gamified applications are the perfect medium for providing health-related interventions. In contrast to traditional methods, using smoking cessation apps including convenient access to therapies in everyday contexts and instant assistance when needed, these digitally gamified applications are now much more widely used than SMS text messaging interventions and quitlines. They incorporate education, motivational strategies, help with quit plans, connections to support groups, quit coaches, and other features [15]. The aim of this systematic review was to systematically assess the effectiveness of digital gamification-based interventions in promoting smoking cessation by evaluating their impact on quit rates, user engagement, behavioral motivation, and adherence compared to traditional or non-gamified methods.

## Review

Methodology

Study Registration and Search Strategy

The study protocol was registered in the International Prospective Register of Systematic Reviews (PROSPERO) database (Registration ID CRD420251136290. A comprehensive electronic search of English-language articles published between January 2015 and July 2025 in PubMed, Scopus, and Web of Science was done. The articles were identified according to the medical subject heading (MeSH) terms using Boolean operators (AND, OR). The search strategy is described in the Table 1.

**Table 1:** Search strategy in the databases

PubMed = 11
("tobacco use cessation"[MeSH Terms] OR "tobacco use cessation"[MeSH Terms] OR (("cessation"[All Fields] OR "cessations"[All Fields]) OR "tobacco use cessation"[MeSH Terms] OR "Smoking"[MeSH Terms] OR "smoking cessation"[MeSH Terms] OR (("cessation"[All Fields] OR "cessations"[All Fields]) AND "Smoking"[MeSH Terms]) OR "giving up smoking"[Title/Abstract] OR "smoking giving up"[Title/Abstract] OR (("smoke"[MeSH Terms] OR "smoke"[All Fields] OR "smoke s"[All Fields] OR "smoked"[All Fields] OR "smokes"[All Fields] OR "Smoking"[MeSH Terms] OR "Smoking"[All Fields] OR "smokings"[All Fields] OR "smoking s"[All Fields]) AND "giving up"[Title/Abstract]) OR "quitting smoking"[Title/Abstract] OR "smoking quitting"[Title/Abstract] OR "stopping smoking"[Title/Abstract] OR "smoking stopping"[Title/Abstract] OR "tobacco use disorder"[MeSH Terms] OR "nicotine"[MeSH Terms]) AND ("tobacco consumption"[Title/Abstract] OR "tobacco habit"[Title/Abstract] OR "smoker"[Title/Abstract] OR "smokers"[Title/Abstract]) AND ("gamification"[MeSH Terms] OR "gamification"[All Fields] OR "gamified"[All Fields] OR "gamification"[MeSH Terms] OR "gamified apps"[Title/Abstract] OR "gamified app"[Title/Abstract] OR (("gamification"[MeSH Terms] OR "gamification"[All Fields]) AND "app"[Title/Abstract]) OR "gamification apps"[Title/Abstract] OR "game-based"[Title/Abstract] OR "game based apps"[Title/Abstract] OR "game based app"[Title/Abstract] OR "gamified application"[Title/Abstract] OR "gamified applications"[Title/Abstract] OR "game based app"[Title/Abstract] OR "game based apps"[Title/Abstract] OR "game based application*"[Title/Abstract] OR (("gamification"[MeSH Terms] OR "gamification"[All Fields]) AND "app"[Title/Abstract]) OR "gamification apps"[Title/Abstract] OR (("gamification"[MeSH Terms] OR "gamification"[All Fields]) AND "application*"[Title/Abstract]))
Scopus = 30
( "tobacco use cessation" OR "tobacco use cessation" OR ( ( cessation OR cessations ) AND "tobacco, smoking ) OR "tobacco use cessation" OR smoking OR "smoking cessation" OR ( ( cessation OR cessations ) AND smoking ) OR "tobacco use cessation" OR "giving up smoking" OR "smoking giving up" OR ( ( smoke OR smoke OR "smoke s" OR smoked OR smokes OR smoking OR smoking OR smokings OR "smoking s" ) AND "giving up" ) OR "quitting smoking" OR "smoking quitting" OR "stopping smoking" OR "smoking stopping" OR "tobacco use disorder" OR nicotine ) AND ( "tobacco consumption" OR "tobacco habit" OR “smoker OR smokers" ) AND ( gamification OR gamification OR gamified OR gamification OR "gamified apps" OR "gamified app" OR ( ( gamification OR gamification ) AND app ) OR "gamification apps" OR game-based OR "game based apps" OR "game based app" OR "gamified application" OR "gamified applications" OR "game based app" OR "game based apps" OR "game based application*" OR ( ( gamification OR gamification ) AND app ) OR "gamification apps" OR ( ( gamification OR gamification ) AND application* ) )
Web of Science = 95
(((((ALL=(gamification)) OR ALL=(gamified app)) OR ALL=(gamified apps)) OR ALL=(game based app)) OR ALL=(game based apps)) OR ALL=(mobile game) AND (((((((((((ALL=(tobacco cessation)) OR ALL=(smoker)) OR ALL=(smokers)) OR ALL=(quit tobacco)) OR ALL=(tobacco use cessation)) OR ALL=(smoking cessation)) OR ALL=(smoking cessations)) OR ALL=(tobacco cessations)) OR ALL=(tobacco use cessations)

Review questions: (1) What is the effectiveness of gamified digital interventions (e.g., mobile apps, games) in promoting smoking cessation compared to traditional or non-gamified approaches? (2) Do gamification-based smoking cessation interventions improve quit rates, reduce cravings, or decrease tobacco use over short-term and long-term follow-up?

Eligibility Criteria and Type of Studies 

This review included English-language full-text articles published between 2015 and 2025 that focused on smoking cessation using game-based interventions, including original studies and clinical trials. In our review, we defined short-term outcomes as those assessed within ≤3 months of follow-up, which primarily capture initial abstinence, motivation, and engagement. Long-term outcomes are defined as those assessed at >3 months, typically up to 6-12 months, which provide stronger evidence of sustained abstinence. Exclusion criteria comprised secondary reports following original publications, inaccessible full texts, unpublished studies, and articles not directly related to the topic. Additionally, studies addressing only general smoking behavior without evaluating cessation outcomes, as well as qualitative studies, case reports, reviews, editorials, and conference abstracts, were excluded. Articles not published in English or those providing insufficient data on smoking cessation outcomes, or where gamification was a minor or unrelated component, were also excluded. The PICO framework applied was as follows:

Population (P): Individuals who smoke and attempting smoking cessation.

Intervention (I): Digital gamification-based interventions for smoking cessation, including mobile applications, serious games, game-based behavioral interventions, online interactive platforms with gamified elements (badges, leaderboards, rewards, challenges).

Comparison (C): (1) No intervention or usual care; (2) traditional tobacco cessation methods (e.g., counselling, nicotine replacement therapy, non-gamified mobile apps); (3) other digital interventions without gamification elements.

Outcome (O): (1) Smoking cessation rates (self-reported or biochemically verified); (2) changes in motivation and intention to quit; (3) psychological outcomes (e.g., reduction in craving, tobacco dependence or anxiety related to quitting); (4) user experience and acceptability of gamification.

Study Selection

**Figure 1:**
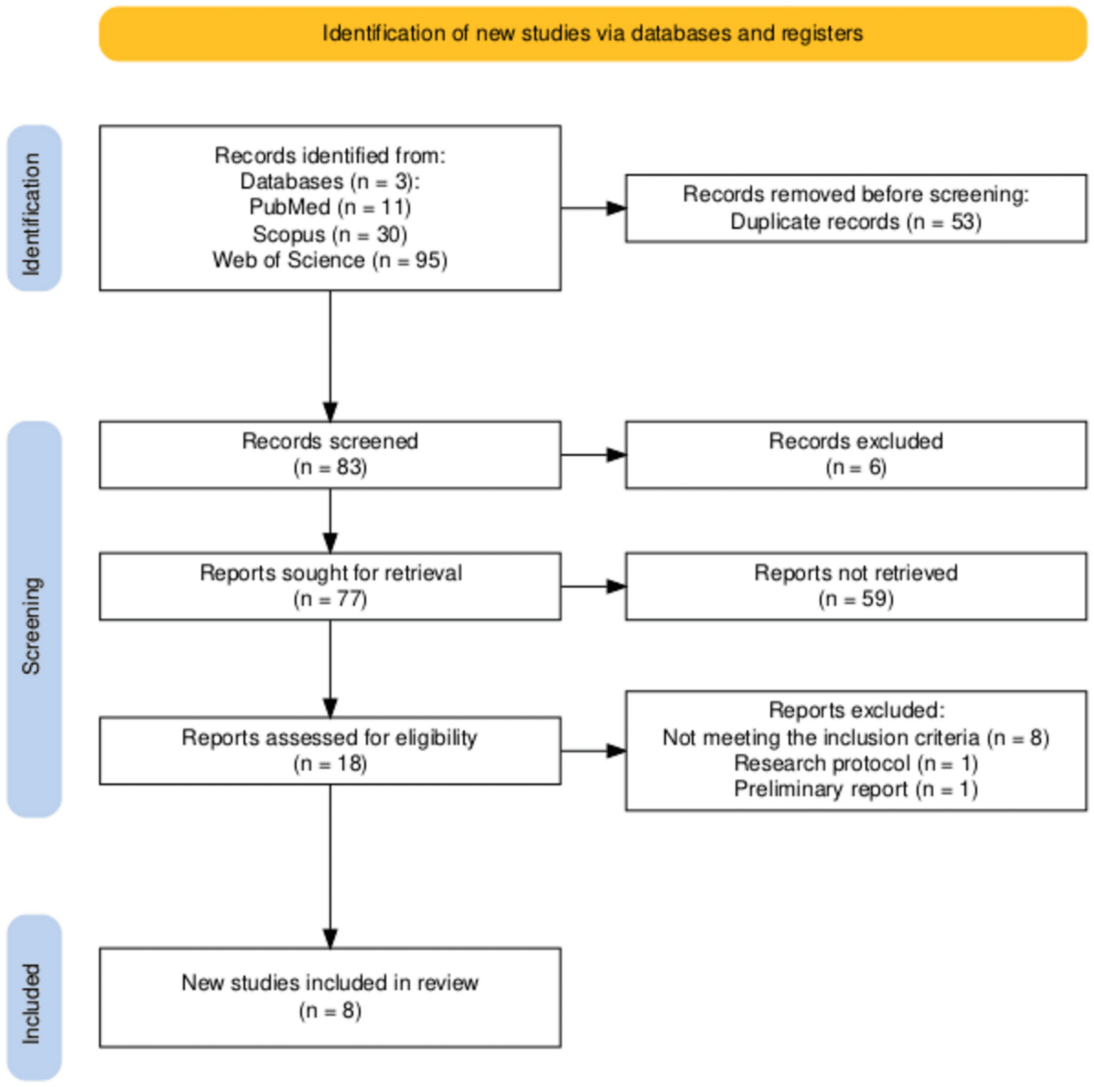
PRISMA flow diagram illustrating the search strategy PRISMA: Preferred Reporting Items for Systematic Reviews and Meta-Analyses.

The methodology of the study adhered to the flowchart of the Preferred Reporting Items for Systematic Reviews and Meta-Analyses (PRISMA) (Figure 1) [16]. Titles and abstracts retrieved from the databases were independently screened for relevance by two reviewers. Any disagreements were resolved through consultation with a third reviewer. Relevant data were then extracted and organized into a table for quality assessment.

Data Extraction

Two independent reviewers used predetermined inclusion and exclusion criteria for screening abstracts and titles. For ultimate inclusion, full-text papers from possibly appropriate studies were evaluated. A third reviewer was consulted and discussion ensued to settle disagreements. A standardized data extraction form was used to collect the following data: study characteristics (author, year, country, study design), intervention details (type of gamification, platform, duration), comparator details, outcome measures, and key findings. The quality of all included studies was evaluated using the National Institutes of Health (NIH) tool, developed by the National Heart, Lung, and Blood Institute (NHLBI), following the checklist criteria for reporting controlled intervention studies [17].

Results

Selection of articles for review

Following the initial search, 136 articles were retrieved from electronic databases. After removing duplicates, 83 articles remained for title and abstract screening. Of these, 18 were assessed for eligibility, and eight met all inclusion criteria. Table 2 presents a summary of the descriptive characteristics of the included studies. 

**Table 2:** Summary of the characteristics of the included studies NRT: nicotine replacement therapy; d: effect size

Authors, year, and name of the journal	Place of study	Sample size	Randomization	Population	Intervention group (IG)	Control group (CG)	Follow-up	Results	Conclusion
White et al., 2024, Journal of Medical Internet Research ^[6]^	United States (U.S.)	Control: 238. Intervention: 241. Total: 479	Stratified by age & smoking intensity	Adults in the U.S. (N = 479) who smoked ≥1 cigarette/day and planned to quit within 7 days	Inner Dragon-a gamified module within the Smoke Free mobile app. It featured a customizable virtual dragon pet, in-game rewards, craving management tools (e.g., breathing exercises, memory game), social features (dragon park), and experience points tied to smoking cessation activities	Received the standard Smoke Free app with evidence-based smoking cessation features but no gamification (e.g., diary, craving log, chatbot, missions)	8 weeks	Tobacco cessation: • No significant difference in self-reported 7-day abstinence (Intervention: 39%, Control: 42.4%) or biochemically verified abstinence at 2 months. • Repeated 1-day abstinence significantly higher in intervention group (17.3% vs 12.4%, p = 0.01). Psychological/User Outcomes: • No significant differences in motivation to quit, user satisfaction, or digital therapeutic alliance. • Program adherence (use of core app features) and engagement metrics were significantly higher in the gamification group. • No serious adverse events were reported	Inner Dragon increased engagement and adherence but did not significantly improve abstinence. Further refinements needed; gamification shows promise to enhance digital cessation tools.
Peek et al., 2021, JMIR Formative Research ^[7]^	Brisbane, Australia	Control: 33. Intervention: 31. Total: 64	Block randomization, stratified by site	Adults aged ≥50 (mean age = 62), current smokers recruited from hospitals in Brisbane, Australia	My QuitBuddy, a freely available smartphone app with gamified features: goal tracking, motivational badges, personalization, peer encouragement via community forums	Access to a smoking cessation website (Quit HQ) offering weekly motivational emails, stories, and Quitline links	3 months	Tobacco cessation (self-reported 3-month abstinence): 13% (IG) vs 6% (CG), not statistically significant. Motivation to quit: Median 6 in IG vs 4 in CG (p = 0.02). Reduction in cigarette use: Trend toward reduction in IG but not statistically significant. User acceptability: Similar user experience ratings ((User Version of the Mobile App Rating Scale (uMARS)) across groups, though slightly better functionality score for app users	The app was acceptable to a subset of older smokers and increased motivation to quit. Larger trials are needed to evaluate long-term effectiveness
Chen et al. 2020, JMIR Mhealth Uhealth ^[9]^	China	Control: 40. Intervention: 40. Total: 80	Computer-generated block randomization	Male smokers in China aged 25–44 recruited via WeChat; mostly daily smokers; majority had previous quit attempts.	Digital gamification via SCAMPI program on WeChat: • Quitting plan setup • Progress calendar • Calculator for savings • Motivational messages • Gamified leaderboard for longest abstinence • Peer support via social platform • Health assessments (lung test, nicotine dependence)	Access to a static page with contacts for standard cessation services (no gamification or interactive content)	6 weeks	Biochemically verified 30-day abstinence: 25% (IG) vs 5% (CG); p = 0.03, self-reported 7-day abstinence: 63% (IG) vs 33% (CG) at 4 weeks; p = 0.007 , 30-day self-reported abstinence: 38% (IG) vs 13% (CG); p = 0.02	SCAMPI was feasible, acceptable, and preliminarily effective. Larger trials with longer follow-up are needed. High user satisfaction was observed
Schlam et al. 2020, Games for Health Journal ^[10]^	Wisconsin, USA	Control: 14. Intervention: 16. Total: 30	Stratified by gender and craving levels	30 adult smokers (mean age ~41 years), recruited in Wisconsin via Facebook, interested in quitting, minimal prior mobile gaming use.	11 commercial mobile games integrated into a custom app (e.g., Solitaire, Temple Run, Quell Memento+) played on a study smartphone. Gamified craving tracking and distraction mechanism (games used as a substitute behavior post-target quit day (TQD))	Received the same nicotine patch and counselling, but no access to mobile games or app post-TQD	4 weeks	Games-on group showed slight craving reduction, games-off group showed increase; 25% carbon monoxide (CO); confirmed abstinence in IG vs 21.4% in CG at 4 weeks; games-on participants played games and reported moderate craving relief (mean 3.22/5)	Games may modestly help manage cravings; further trials with larger samples are warranted. Games were rated helpful but underused, possibly due to limited phone/game accessibility
Scholten et al., 2019, Development and Psychopathology ^[11]^	Netherlands (Radboud University)	Control: 72. Intervention: 72. Total: 144	Blocked randomization	Young smokers (age 16-26), Netherlands, recruited through schools and social media. Mostly moderate nicotine dependence	HitnRun: A mobile runner-style game using Go/No-Go training, personalized prompts, team-based challenges, Google Hangouts peer interaction, cooperative gameplay, daily craving distraction	Received a psychoeducational brochure on quitting smoking (Trimbos Institute), no gamification or social interaction	4 weeks intervention + 3-month follow-up	Both groups showed significant reductions in weekly smoking, but no difference between brochure and game groups in abstinence or smoking behavior. Dose-response effect in game group: high game users smoked significantly less than low users. Peer engagement and game evaluation positively correlated with smoking reduction	While HitnRun was not superior to a brochure overall, greater game engagement led to better outcomes. Peer-based mobile games show promise and merit further refinement and testing
Krebs et al. 2019, JMIR Mhealth Uhealth ^[13]^	New York, United States	Control: 18. Intervention: 20. Total: 38	Stratified by age, 1:1 randomization	English-speaking adult cancer patients (recent diagnosis, undergoing surgery), current smokers (past 30-day use), recruited from a hospital-based tobacco program at MSKCC, New York. Mean age: 57	QuitIT: Tablet-based coping skills game involving interactive episodes, urge tracking, character navigation, and gamified reinforcement (points, badges, scenarios). Reinforces real-world coping strategies via digital simulations	Standard Care (SC): • 4 sessions of telephonic/bedside counseling • Print education • Pharmacotherapy support as needed (e.g., NRT, medications) • No app, no game elements	1 month	Tobacco abstinence (biochemically verified): 30% (IG) vs 18% (CG). Self-efficacy/confidence to quit: increased in IG (d = 0.25). Intention to stay abstinent: significantly higher in IG (d = 1.03). User satisfaction: 63-75% reported game was helpful/fun/relatable. Adherence: only 40% played the game. No serious adverse events	The QuitIT game showed promising trends in improving abstinence and motivation, but feasibility was low due to poor engagement and limited tablet use. Recruitment was challenging post-hospitalization; further optimization and training needed for larger-scale trials
Peiris et al., 2019, JMIR Mhealth Uhealth ^[14]^	Australia	Control: 24. Intervention: 25. Total: 49	Computer-based randomization (minimization algorithm stratified by sex, age, and addiction level)	Aboriginal and Torres Strait Islander current smokers aged ≥16 in Australia, motivated to quit within one month; recruited from health services and Quitline	Can’t Even Quit app: Personalized quit plan, motivational texts, challenge feature (gamified competition), in-app tracking and support features	Access to available cessation services (e.g., Quitline, local support) but no app or gamification	6 months	CO-verified abstinence: 1 participant (4.5%) in IG; none in CG. Quit attempts: Similar between groups. App engagement: Low (2.9 sessions/month, 6.3 mins/session). Qualitative feedback suggested need for enhanced gamification & social connectivity	App was acceptable but low engagement limited impact. Recruitment challenges affected power. Participants favored more gamified and social features
Baskerville et al., 2018, JMIR Mhealth Uhealth ^[15]^	Canada	Control: 779. Intervention: 820. Total: 1599	Computer-generated	Canadian young adult smokers (aged 19-29), daily smokers intending to quit in 30 days, recruited via online ads	Crush the Crave (CTC) smartphone app: personalized quit plan, health tracking (money saved, health gains), push notifications, rewards/milestones, social media sharing (Facebook/Twitter), coping tools for cravings, Quitline information	On the Road to Quitting (OnRQ) self-help guide: Digital and printable format. Same behavioral content as app-no gamification, app-based interactivity, or social features	3 and 6 months	No significant difference in abstinence: • Continuous abstinence (6 months): 7.8% (CTC) vs 9.2% (control) • 30-day abstinence: 14.4% (CTC) vs 16.9% (control) • Higher satisfaction and helpfulness scores in control group • Quit attempts and cigarettes/day: no significant difference	Although CTC was an effective method for abstaining, it didn't outperform self-help materials for motivated young adults. To increase user satisfaction and boost the efficacy of digital smoking cessation therapies, more study is required

For subsequent data extraction, full-text articles were selected based on the predefined inclusion and exclusion criteria for detailed analysis. The reviewed articles identified five major domains reflecting the effectiveness of digital gamification in smoking cessation: (1) user engagement and adherence [6,13,11,14]; (2) effectiveness in tobacco abstinence [9,13,10,15]; (3) psychological/motivational outcomes [7,13,11]; (4) acceptability and user satisfaction [7,10,9,13]; and (5) limitations and challenges in implementation [14,13,15].

User Engagement and Adherence

Gamification has shown strong potential to improve user engagement. Features such as virtual pets (e.g., Inner Dragon), leaderboards (SCAMPI), and personalized challenges (e.g., Can’t Even Quit) encouraged participants to interact more frequently with the apps. However, higher engagement did not consistently lead to higher abstinence rates. Some studies demonstrated a dose-response relationship, those who engaged more tended to reduce smoking more, but others saw low usage despite high initial acceptability, especially when accessibility or usability was limited [6,13,11,14].

Effectiveness in Tobacco Abstinence

The effectiveness of gamified interventions in achieving abstinence was mixed. A few studies reported significant improvements like SCAMPI led to a fivefold increase in biochemically verified 30-day abstinence compared to controls. QuitIT game-based intervention study showed a higher quit rate and intention to remain abstinent. Other studies with game-based interventions like Crush the Crave and HitnRun did not outperform traditional methods or brochures, although they indicated promise with higher app use. This indicates that gamification may support cessation, but its standalone efficacy is inconsistent and often influenced by engagement levels, individual motivation, and context [9,10,13,15].

Psychological/Motivational Outcomes

Gamified features positively influenced motivation, confidence, and intention to quit: Peek et al. found significantly higher motivation scores in the intervention group [7], Krebs et al. reported increased self-efficacy and stronger intention to abstain among users of the gamified app [13], Scholten et al. linked peer engagement and game satisfaction with smoking reduction [11].

Acceptability and User Satisfaction

Most users found gamified apps to be acceptable, engaging, and easy to use. Satisfaction was particularly high in studies where apps were well-integrated with daily behavior (e.g., WeChat-based SCAMPI) and games were fun and relatable (e.g., QuitIT, Inner Dragon). However, technical challenges, device incompatibility, or limited customization often affected usability, underlining the importance of user-centric design [7,13,9,10].

Limitations and Challenges in Implementation

Gamified interventions face practical barriers, poor engagement often stemmed from inadequate interactivity, low digital literacy, or cultural mismatch; some settings (e.g., post-surgical patients in Krebs et al.) were not conducive to app use; gamification alone may not suffice; integration with behavioral support and personalization appears necessary for sustained outcomes [14,13,15].

Quality Assessment

**Figure 2:**
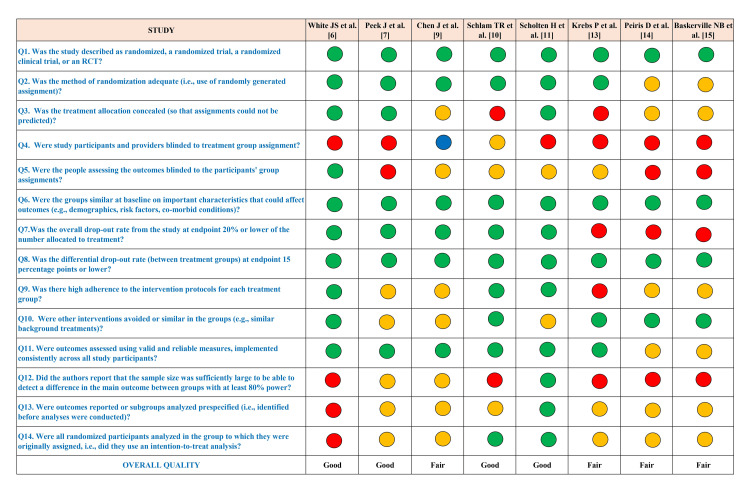
Quality assessment of included studies using the NIH checklist NIH: National Institutes of Health

Figure 2 illustrates the quality ratings of the randomized controlled trials. Green circles represent “yes,” red circles represent “no,” while yellow, grey, and blue circles indicate “not reported,” “cannot determine,” and “not applicable,” respectively.

Discussion

Through this systematic review, we found consistent evidence that gamification in smoking cessation interventions enhances motivation and self-efficacy, but direct evidence for improved long-term quit rates remains limited. Our findings align closely with recent observational and interventional studies, while also highlighting important contrasts and evidence gaps.

Across many studies, gamification consistently increased user engagement and improved key psychological factors. For example, Rajani et al. showed that people who used gamification features more often in quit-smoking apps gained higher motivation and confidence, with goal-setting and progress tracking being the most useful features, while social-sharing options were rated the least helpful [18]. Another study showed that even smokers not planning to quit responded well to short-term gamified challenges, setting goals and staying engaged [19]. A different study on the NoFumo+ CBT-based app found that both experts and smokers found it easy to use and suggested adding more gamified features [20]. In a trial with adolescents, Nyman et al. found that gamification improved confidence in refusing cigarettes, especially among younger participants and those influenced by parents or peers who smoked [21]. These results support that gamification strengthens important psychological pathways that help people quit.

However, while gamification clearly improved short-term outcomes like engagement, enjoyment, and motivation, most studies did not provide strong evidence on long-term quitting confirmed by biological tests. Edwards et al. showed that game-based designs can be fun and practical but did not prove lasting quit rates [22]. García-Pazo et al. also found good usability but stressed the need for larger clinical trials [20]. Youth-focused programs showed strong effects on refusal confidence but did not track quitting outcomes in adults. Early work with VR also suggested gamification increases enjoyment and commitment, but solid outcome data are missing.

Overall, the studies show that gamification boosts motivation and self-confidence for quitting. Still, proof that it leads to permanent quitting is weak. Our review suggests focusing on useful gamification features like goal-setting, progress dashboards, and personalized feedback, while being cautious with social-sharing tools that may not connect well with smokers. Importantly, gamification seems especially promising for smokers with low motivation, a group often missed by traditional programs. Future studies should test gamified designs in large, well-structured trials with at least 6-12 months of follow-up and biochemical confirmation of quitting.

This review has certain limitations. First, only studies published in English between 2015 and 2025 were included, which may have led to language and publication bias. Second, the small number of eligible studies (n = 8) and their heterogeneity in terms of study design, intervention type, population, and follow-up periods limit the generalizability of findings. Third, many included studies relied on self-reported smoking outcomes without biochemical verification, raising the possibility of reporting bias. Additionally, engagement levels varied widely, and most interventions lacked long-term follow-up beyond 6-12 months, making it difficult to draw firm conclusions about sustained abstinence. Finally, the rapid pace of technological advancement means that some interventions may already be outdated, highlighting the need for continuous evaluation of newer gamification strategies.

## Conclusions

Gamification shows strong potential as an innovative approach to enhance user engagement, motivation, and self-efficacy in smoking cessation. While certain interventions such as SCAMPI and QuitIT demonstrated promising short-term effects, the overall evidence for long-term abstinence remains limited and inconsistent. Gamification seems to work best when it is used along with traditional quit methods such as counseling or nicotine replacement. Future studies should test these apps in larger groups, for longer periods, and confirm results with medical checks. It is also important to make sure the apps are accessible, culturally suitable, and safe for users’ data. Gamification may not yet replace regular quit programs, but it is a promising and engaging add-on to help more people quit smoking.
